# When a parent dies – a systematic review of the effects of support programs for parentally bereaved children and their caregivers

**DOI:** 10.1186/s12904-017-0223-y

**Published:** 2017-08-10

**Authors:** Ann-Sofie Bergman, Ulf Axberg, Elizabeth Hanson

**Affiliations:** 1 Department of Social Work, Swedish Family Care Competence Centre, Linnaeus University, SE-351 95 Vaxjo, Sweden; 2 Department of psychology, University of Gothenburg, SE-40530 Gothenburg, Sweden; 3 Swedish Family Care Competence Centre, Linnaeus University, SE-391 82 Kalmar, Sweden; 4 University of Sheffield, Sheffield, UK

**Keywords:** Bereavement, Grief, Parental death, Death, Dying, Bereavement support, Intervention, Evaluation

## Abstract

**Background:**

The death of a parent is a highly stressful life event for bereaved children. Several studies have shown an increased risk of mental ill-health and psychosocial problems among affected children. The aims of this study were to systematically review studies about effective support interventions for parentally bereaved children and to identify gaps in the research.

**Methods:**

The review’s inclusion criteria were comparative studies with samples of parentally bereaved children. The focus of these studies were assessments of the effects on children of a bereavement support intervention. The intervention was directed towards children 0–18 years; but it could also target the children’s remaining parent/caregiver. The study included an outcome measure that dealt with effects of the intervention on children. The following electronic databases were searched up to and including November 2015: PubMed, PsycINFO, Cinahl, PILOTS, ProQuest Sociology (Sociological Abstracts and Social Services Abstracts). The included studies were analysed and summarized based on the following categories: type of intervention, reference and grade of evidence, study population, evaluation design, measure, outcome variable and findings as effect size within and between groups.

**Results:**

One thousand, seven hundred and-six abstracts were examined. Following the selection process, 17 studies were included. The included studies consisted of 15 randomized controlled studies, while one study employed a quasi-experimental and one study a pre-post-test design. Thirteen studies provided strong evidence with regards to the quality of the studies due to the grade criteria; three studies provided fairly strong evidence and one study provided weaker evidence.

The included studies were published between 1985 and 2015, with the majority published 2000 onwards. The studies were published within several disciplines such as psychology, social work, medicine and psychiatry, which illustrates that support for bereaved children is relevant for different professions. The interventions were based on various forms of support: group interventions for the children, family interventions, guidance for parents and camp activities for children. In fourteen studies, the interventions were directed at both children and their remaining parents. These studies revealed that when parents are supported, they can demonstrate an enhanced capacity to support their children. In three studies, the interventions were primarily directed at the bereaved children. The results showed positive between group effects both for children and caregivers in several areas, namely large effects for children’s traumatic grief and parent’s feelings of being supported; medium effects for parental warmth, positive parenting, parent’s mental health, grief discussions in the family, and children’s health. There were small effects on several outcomes, for example children’s post-traumatic stress disorder (PTSD) symptoms, anxiety, depression, self-esteem and behaviour problems. There were studies that did not show effects on some measures, namely depression, present grief, and for the subgroup boys on anxiety, depression, internalizing and externalizing.

**Conclusions:**

The results indicate that relatively brief interventions can prevent children from developing more severe problems after the loss of a parent, such as traumatic grief and mental health problems. Studies have shown positive effects for both children’s and remaining caregiver’s health. Further research is required including how best to support younger bereaved children. There is also a need for more empirically rigorous effect studies in this area.

## Background

In stable developed nations about three to 4 % of children are affected by the loss of a parent through death prior to the age of 18 [1]. The loss of one or both parents can be associated with a higher vulnerability for children, both from a short and long term perspective. Several studies have shown an increased risk of mental health problems and threats to emotional well-being for affected children, such as anxiety, depression and a perceived lack of control over what happens in one’s life [1–5]. The death of a parent has also been linked to increased somatic symptoms and development of stress sensitivity [2, 6, 7]. Scandinavian studies have revealed that the death of a parent in childhood or adolescence is associated with an increased mortality risk during childhood, adolescence and into early adulthood [8, 9]. Parental death in childhood is also associated with an increased long-term risk of suicide [10]. A child’s problems post bereavement may also appear in school as concentration difficulties or behavioural problems [1, 2]. A longitudinal study by Brent et al. [11] reported that suddenly (e.g. unexpected deaths) bereaved youths had lower competence than non-bereaved youths in the areas of work and future education planning.

After the death of a parent some children live with their remaining parent, while other children live with another person, for example a stepmother, stepfather, grandparent, aunt, uncle, sibling, foster parent, adoptive parent. In this article we use the term *caregiver* to refer to a surviving parent or another significant other who takes on board a parental role.

The death of a parent is a highly stressful life event for children. While children at this time are in significant need of support, the inverse can happen because of changes in the family situation and family roles post bereavement. In some cases, the children’s remaining parent/caregivers are struggling with their own grief and may experience psychological difficulties themselves. As a result, it can be a challenge for them to provide sufficient support for the children. The remaining parent must also deal with additional stressors of being a single parent and the sole provider of support, while simultaneously coping with the loss of their partner [12]. For the children, this can mean reduced time, attention and support from their remaining parent/caregiver.

Some children, who lose a parent under traumatic circumstances (such as deaths due to violence, suicide, accident, war or disaster), may suffer from traumatic grief. In some instances, death from natural anticipated causes may also result in traumatic grief, if the child’s experience of the death was shocking. The children can re-experience the traumatic event through intrusive memories, thoughts and feelings. The distress leads to avoidance of trauma and loss reminders. The child may avoid thinking or talking about the deceased parent, places and activities associated with the parent. The traumatic experience often complicates the children’s grieving process [13]. After the loss of a parent children can also develop prolonged grief disorder, a disorder that includes a persistent and disruptive yearning [14]. The child may also have difficulties in accepting the parent’s death and difficulties in moving on in their own lives. The child may also experience feelings of bitterness, and a sense that life is meaningless as part of the syndrome detachment [14].

When a parent dies, the children and the remaining parent/caregiver may need advice and support in their grieving process from a health care professional, in order that their mental health needs are met and so that they can continue their development in a positive direction. However, a key question in the field is what kinds of support are most effective for the children and their caregivers?

While previous reviews in the field have had a broader focus, namely treatment effects for children who have lost a “loved one”, such as a family member, grandparent, relative or friend [15–17], the review presented in this paper focuses on the effects of support interventions for children who are parentally bereaved. The rationale for this in-depth focus is that it is recognised that there are distinct difficulties for children losing a parent and caregiver, as this is often the person that previously was central in the provision of love, security and daily care. This closer relationship means higher impact for the child and heightened feelings of loss and bereavement [2].

In this paper, we present findings from a systematic review of empirical studies evaluating the effectiveness of supportive interventions for children when a parent or caregiver dies. In so doing we may identify gaps in the research. Our research questions are: Which support interventions have been evaluated that focus on effects for children? What is known about the effects of support interventions for the children? What are the needs for further research in the field?

## Method

Our review inclusion criteria were studies:

1. Published in English or Scandinavian languages.
2. Sample populations of parentally bereaved children to 18 years of age.
3. Evaluating the effects of bereavement interventions for the children. Family programs were included if children were included in the intervention and the evaluation.
4. Those were randomized controlled design, quasi experimental design or pre-post-test design.

Working with an information specialist at the National Board of Health and Welfare Sweden, a systematic literature search was undertaken in April 2013 to identify relevant references. Six electronic databases were searched, PubMed, PsycINFO, Cinahl, PILOTS, ProQuest Sociology (Sociological Abstracts and Social Services Abstracts). An updated database search was undertaken in November 2015 to identify studies of bereavement support interventions. We used search terms including: bereavement; grief; parental death; parental bereavement; parentally bereaved child; parentally bereaved youth; parental loss; dying parents; loss of a parent; childhood bereavement; children’s grief; grieving child; combined with search terms related to interventions and evaluation (For full details please contact the first author). Reference lists in the identified literature and previous reviews in the field were also scanned to locate additional relevant studies.

During the selection of studies *The Cochrane Handbook for Systematic Review of Interventions* (http://handbook.cochrane.org/) was used as a guide. All retrieved studies were reviewed independently by two of the authors. In the initial screening stage, only studies that were obviously irrelevant were excluded. In cases where the researchers made different selections, the studies were included for further review by two authors reading the full paper. In the case of disagreement, two researchers discussed the studies until consensus was reached. Studies were excluded for the following reason: the study population in the evaluation was small, i.e. studies with a population of less than 30 participants.

The evidence was graded according to the rigour of the study design and analysis. We used the same grading criteria as Harding & Higginson [18] and Hudson et al. [19] in their reviews of intervention studies [20]. The assessment and grading criteria are shown in Table 1.

**Table 1:** Grade Criteria

Grade I (Strong evidence) RCTs or review of RCTS IA Calculation of sample size and accurate standard definition of appropriate outcome variables IB Accurate and standard definition of appropriate outcome variables IC Neither of the above
Grade II (Fairly strong evidence) Prospective study with a comparison group (non-randomized controlled trial, good observational study or retrospective study that controls effectively for confounding variables) IIA Calculation of sample size and accurate, standard definition of appropriate outcome variables and adjustment for the effects of important confounding variables IIB One or more of the above
Grade III (Weaker evidence) Retrospective or observational studies IIIA Comparison group, calculation of sample size, accurate and standard definition of appropriate outcome variables IIIB Two or more of the above IIIC None of these
Grade IV (Weak evidence) Cross-sectional study, Delphi exercise, consensus of experts

Cancer Guidance Subgroup of the Clinical Guidance Outcomes Group. Improving outcomes in breast cancer – the research evidence. Leeds: NHS Executive, 1996 [20]

### Data analysis

Our analysis of the included studies were grouped in a table based on the following categories: type of intervention, reference (comparison), grade of evidence, study population, evaluation design, measure, outcome variable and findings as effect size within (at baseline and follow-up) and between study comparison groups.

For any ordinal or continuous variables, to be able to calculate effect size even when a means and standard deviation were not reported in studies, the standardized mean difference effect size for within-subjects design was used, which is referred to as Cohen’s d_z_. The effect size estimate Cohen’s d_z._ can be calculated directly from the t-value using the formula ${d}_z=t/\sqrt{n}$. A commonly used interpretation of Cohen’s d is that value of 0.2 can be considered a small effect, 0.5 a medium effect and 0.8 a large effect [21].

The Common Language effect size (CL) [22] is also reported. The CL is also known as the probability of superiority [21], represents the probability in percent that a randomly selected person will score a different observed measurement post- than pre intervention, after controlling for individual differences. In addition when possible, the effect size of difference between groups was calculated (dm) using a method proposed by Morris in which effect size is calculated on the mean pre-post change in the treatment group minus the mean pre-post change in the control group, divided by the pooled pre-test standard deviation [23]. For categorical data, Chi-squared tests were made. Phi is reported as the effect size proposed by Fritz and colleagues using the formula $\varphi =\sqrt{\frac{\chi 2}{N^{\prime }}}$ [24]. A value of 0.1 is considered a small effect, 0.3 a medium effect and 0.5 a large effect.

## Results

**Fig. 1:**
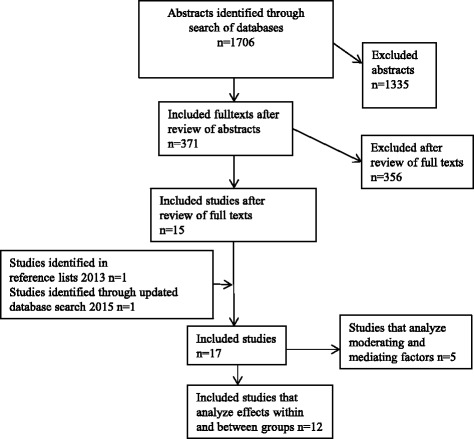
Search flow diagram

The total number of citations identified in the database searches in April 2013 was 1706. Following the screening process, 371 references were selected for further review of full texts. After examination of full texts, a total of 15 studies were identified that evaluated the effectiveness of bereavement interventions with parentally bereaved children [25–39]. We identified an additional study from checking of the reference lists [40]. The number of citations generated in the updated search in November 2015 was 921. Of these five citations were reviewed in full texts. An additional relevant study was identified [41], resulting in a total of 17 selected studies for the review, see Fig. 1 below.

### Included studies

The included 17 studies were published between 1985 and 2015, the majority, 13 were published after 1999. Most studies were conducted in the United States [26, 27, 29–39, 41]; two in England [25, 40], and another was an international collaborative study involving Iran, UK and Norway [28].

### Quality of included studies

The studies differed; they were based on different study designs, contained a variety of outcome measures and varied in quality. According to our quality grading criteria (Table 1) [18–20] 13 studies provided strong evidence. These studies were randomized controlled trials involving validated measures. Three studies provided fairly strong evidence and one study provided weaker evidence [18–20]. Two of the included bereavement interventions were evaluated with a population of more than 100 children. Namely, “The Parent Guidance Program” [26] and “The Family Bereavement Program” [27, 29, 30, 33–35, 37, 39, 41]. One of the interventions, Family Therapy sessions, was tested in two papers [25, 40] and one, The Family Bereavement Program, in as many as ten papers [27, 29, 30, 33–35, 37–39, 41].

### Study design

One study employed a quasi-experimental design [31] and one study had a pre-test/post-test design [36], the others were randomized controlled trials. What the intervention was compared with varied: no intervention [25, 28, 40]; delayed treatment [31, 32]; a telephone support intervention [26]; and a self-study program [27, 29, 30, 33–35, 37–39, 41].

The core concepts addressed in the outcome measures were:

- Children’s health, in particular their mental health (internalization, externalization, coping, stress, cortisol-levels)
- Children’s grief symptoms (traumatic grief, problematic grief)
- Children’s behaviour and school problems
- Children’s self-esteem
- Children’s concepts of death and communication about the deceased parent
- Parenting (communication, caregiver-child relationship, parental warmth, acceptance, consistent discipline)
- Caregiver’s mental health

Fifty different outcome measures were employed. We present the most commonly reported outcomes in the included studies which focus on children’s health, behaviour, grief, self-esteem, parenting factors and caregivers’ mental health [42–54] (see Table 2 below).

**Table 2:** The most common outcome measures employed in the included studies

Children’s health and behaviour	Child Behaviour Checklist (CBCL) [42] Children’s Depression Inventory (CDI) [43] Youth Self-Report (YSR) [42] Children’s Manifest Anxiety Scale-Revised (R-CMAS) [44]
Children’s grief	The Extended Grief Inventory (EGI) [51] Intrusive Grief Thoughts Scale (IGTS) [52] Adapted Inventory of Traumatic Grief: Symptoms of prolonged grief disorder (ITG) [45] Traumatic Grief Inventory for Children (TGIC) [46] The Texas Revised Inventory of Grief (TRIG) [47]
Children’s self-esteem	The Self Perception Profile for Children (SPPC) [53]
Parenting factors	Children’s Reports of Parental Behaviour Inventory (CRPBI) [48] Parent Perception Inventory (PPI) [54]
Caregiver’s mental health	Beck Depression Inventory (BDI) [49] Psychiatric Epidemiology Research Interview (PERI) [50]

### Interventions

A key research question for this review is: What types of support interventions were evaluated in the studies? We found studies varied in their theoretical under-pinning and aim. They also took various forms: group interventions for the children [28, 36], family interventions [25, 27, 29, 30, 32–35, 37–41], parental guidance [26], and camp activities for children [31].

Some interventions were designed based on resilience, risk and protective factors for parentally bereaved children [27, 29, 30, 32–35, 37–39, 41]. Others were based on theory of trauma and/or the grieving process [28, 31]; psycho-education [26]; psychodynamic theory [36]; and attachment theory [25, 40]. To a large extent, the interventions were directed towards children at an early stage in their grief process. “The Family Bereavement Program” and “The Parent Guidance Program” were explicitly intended to be preventive interventions [26, 33]. However, the intervention “Writing for recovery” was directed at refugee children with high symptoms of traumatic grief [28]. For some of the refugee children, many years had passed since their parents died.

In three of the studies, the interventions were primarily directed at the bereaved child in the form of support groups and/or camp activities [28, 31, 36]. The intentions in these studies were: to provide emotional support; to normalize the children’s experiences after the loss; to provide a safe environment where the child can express emotions and thoughts; to facilitate the child’s grieving process and to aim to improve the child’s physical and mental health. For further description of the interventions, see Table 3.

**Table 3:** Intervention description

Study	Intervention description
Schilling et al. 1992 [36] (USA)	Group intervention, “Bereavement groups for inner-city children” Groups consisting of 6–8 children, age 6–12 years 12 sessions divided into 3 phases, each of 4 sessions *Opening phase*: rules of confidentiality, conduct, purpose of the group; focus on the children’s relationship to the deceased and the impact of the loss on their family; sharing experiences related to death; supportive environment; normalizing bereavement issues *Working phase*: focus on children’s feelings of sadness, anger, ambivalence related to the loss; demystifying irrational thoughts and fears about the death; identifying and expressing painful feelings *Ending phase*: the termination of the group as another loss; encourage children to utilize their family as support system; children were reassessed to determine the need for further treatment
McClatchey et al. 2009 [31, 55] (USA)	Group intervention, camp activities, “Camp MAGIC” Groups consisting of 5–8 children, separate groups for children age 7–11 and 12–17 years *Camp activities:* such as ropes course, canoeing, archery, interacting with new friends *Counseling sessions*: 6 counseling sessions during a weekend (Friday-Sunday) *Focus on*: trauma experience; trauma and loss reminders; post-traumatic adversities; interplay of trauma and grief; resumption of developmental progression Grief-oriented tasks and cognitive behavioural aspects such as exposure, cognitive restructuring, stress inoculation techniques *Activities:* related to grief processing such as creation, play, puppetry show, memorial service Psychoeducational workshop for parents about children’s grieving process
Kalantari et al. 2012 [28] (Iran/UK/Norway)	Group intervention “Writing for recovery” Intervention for children age 12–18 years 6 sessions in school during three consecutive days, each day consists of two 15-min sessions Writing about traumatic experiences to decrease negative thoughts and feelings *Writing sessions*: Progress from unstructured expressive writing about innermost feelings and thoughts about the traumatic event/loss, to more structured writing where children reflect on what they would have given as advice to another in the same situation as themselves. In the last writing session children are asked to imagine that 10 years has passed and they look back and think about what they have learned from their experience
Black & Urbanowicz 1985 [40]; Black & Urbanowicz 1987 [25] (UK)	Family intervention, family therapy sessions, with children age 0–16 years and their families 6 family therapy sessions spaced at 2–3 weeks intervals, in the families’ homes *Focus on*: help with emotional and practical problems arising from bereavement; promote mourning in both children and surviving parent; improve communication between children and parent; improve communication about death; encourage children to talk about the dead parent and their feelings of loss and grief; encourage expression of grief in the family Separate sessions for parents alone to enable him/her to talk about his/her own grief, anger, needs
Christ et al. 2005 [26] (USA)	Intervention directed to the well parent and the family when a parent has cancer and is terminally ill, “The Parent Guidance Program” Families with children age 7–17 years 6 or more 60–90 min therapeutic sessions during the terminal stage of the parents illness and 6 or more sessions after the parents death, including meetings with parent(s), children and family *Focus on*: to affect the children’s adjustment to the loss by enhancing the surviving parents ability to sustain competence in providing support and care or the children; provide an environment in which the children feel able to express painful or conflicting feelings, thoughts, fantasies about the loss; maintain consistency and stability in the children’s environment; support to parents in their own grief work in order to enhance their capacity to function effectively during the family crisis; problem solving around the immediate crisis; communication about illness, loss, grief, reactions; future planning for the family
Sandler et al. 1992 [32] (USA)	Family intervention “The Family Bereavement Program” Intervention for families with children age 7–17 years Program including a total of 13 sessions, consisting of a family grief workshop and a family adviser program Family grief workshop, with 8 bereaved families per session *Focus on*: to fulfil the perceived needs of bereaved families to meet with other families who have similar experiences; to improve warmth in the parent-child relationship; improve communication about grief experiences Family adviser program, 12 sessions, including 6 individual sessions for parents and 6 family sessions *Focus on*: parental support; provide emotional support; decrease parental demoralization; increase warmth of the parent-child relationship; increase positive exchanges between family members; increasing quality time between parent and child; communication in the family; planning of stable events; helping improve coping with stressful family events
Sandler et al. 2003 [33]; Schmiege et al. 2006 [37]; Tein et al. 2006 [39]; Sandler et al. 2010 [34]; Sandler et al. 2010 [35]; Luecken et al. 2010 [29]; Hagan et al. 2012 [27]; Schoenfelder et al. 2013 [38]; Luecken et al. 2014 [30]; Schoenfelder et al. 2015 [41] (USA)	Family intervention “The Family Bereavement Program” Intervention for families with children age 8–16 years Program including a total of 14 sessions, consisting of 12 sessions in separate groups for caregivers, children and adolescents Four of these include conjoint activities for children and caregivers. The program also include 2 individual family meetings Groups consisting of 5–9 children, separate groups for children age 8–12 and 12–16. Sessions for caregivers *Focus on*: improving positive caregiver-child relationship; positive parenting; effective discipline strategies; coping with grief; talking to children about grief; increase positive activities; reduce children’s exposure to negative events; family routines; family time; one on one time; communication; listening skills; decrease caregiver mental health problems Sessions for children *Focus on:* improving caregiver-child relationship; positive coping; coping efficacy; control-related beliefs; self-esteem; reduce negative appraisals for stressful events; provide opportunities for expression and validation of grief-related feelings; encouraging sharing of feelings with caregivers; individual goals selected by the children

In the majority of the included studies, the interventions were directed at both the child and their remaining caregiver [25–27, 29, 30, 32–35, 37–41]. The intentions in the included studies were: to provide support for the children and their caregivers; to improve family communication and the caregiver–child relationship; to facilitate participants’ grieving process; to improve their health; strengthen parenting; increase stability and predictability for the children; and to reduce the occurrence of negative events among the children (see Table 3).

In general, the interventions were brief. The shortest program was “Writing for recovery”, involving two 15-min sessions in school during three consecutive days, each day consisted of two sessions (i.e. six 15-min sessions and a total of 90 min) [28]. The camp-based program “CampMAGIC” was delivered over a weekend [31, 55]. The longest, “The Parent Guidance Program” lasted a year, it began when the parent was ill, and continued during the terminal illness and at least 6 months after the parent’s death [26]. It involved at least six sessions during the terminal illness and six after the parent had died. The other interventions were based on a total of 6–14 sessions (see Table 3 for more details).

All interventions were professionally led, in most cases by social workers or counsellors with extensive experience of working with child guidance, grief or psychiatry. The highest educational attainment of professionals were those who led “The Family Bereavement Program”, who had at least a master’s degree [34]. In several studies the intervention leaders received supervision in the implementation of the support program [26, 32, 33, 36].

### Study population

The included interventions in this review were directed at children from school age up to 18 years of age. This is with the exception of two studies where younger children (0–16) were involved in family therapy sessions [25, 40]. Most of the studies concerned children who had experienced a parental death from a range of causes, namely illness, accident, suicide or homicide [25, 27, 29, 30, 32–41]. Commonly parents died because of an illness (65–82%), thereafter due to an accident (15–20%) or suicide/homicide (10–14%). In most studies there was a lack of information about what kind of illness the parent suffered from, where there was information, diseases included those of the heart and cancer [25, 32, 40]. One study compared intervention effects for children who had lost a parent to expected versus unexpected deaths [31]. One study focused on children during their parent’s terminal cancer illness as well as after the parent’s death [26]. Finally one study focused on support directed at refugee adolescents who had lost their parents in war [28]. Except for this evaluation directed at refugee children from Afghanistan, the majority of included studies had samples that were diverse in ethnicity, including for example Caucasian, Hispanic, African American, Native American, Asian/Pacific and other ethnicities [33].

In the studies, the most common deceased parent was the child’s father with the remaining caregiver being the mother. In two of the studies, women as remaining caregivers were over-represented as participants in the study populations [32, 36]. In one study 86% of the deceased parents were fathers and 14% mothers [32]. In another study, fathers as remaining caregivers only represented 5 % of the sample [36].

### Effectiveness of the interventions

Another key research question for this review was: What is known about the effects of support interventions that are targeted at/or include support for parentally bereaved children? The included studies were analysed and summarized in a matrix. The results are presented in table form (see Table 4 below). There were 12 studies that analysed effects within and between trial arms, while five studies analysed moderating and mediating factors. The latter are excluded from the analysis of effects in Table 4, but are nevertheless informative and are therefore included in the article. Our focus is on comparing differences between groups, but we have also chosen to present results within groups in Table 4, as this may be relevant from a benchmarking perspective, both for researchers and clinicians [56]. The results from the analyses of included studies revealed positive effects of the support interventions both for the children and their remaining caregivers in several areas.

**Table 4:** Study effects within and between treatment groups

Interventions directed to the bereaved children													
Intervention	Reference and grade of evidence	Study population	Evaluation design	Measure	Outcome variable	Effect size TG			Effect size CG			Effect size between groups	
						sig	d_z_	CL %	sig	d_z_	CL %	sig	d_m_ (ϕ)
“Bereavement groups for inner-city children”	Schilling et al. 1992 [36]	38 children (age 6–12)	Pre-test/post-test-design	BID	Depr. (Parent) Depr. (Child)	.26 .29	−.22 .21	−59 58	na na	na na	na na	na na	na na
	IIIC		Evaluation: post treatment	ATCD	Attitudes and Concepts of Death	.01	.52	70	na	na	na	na	na
Grief camp “Camp MAGIC” (CG) delayed treatment	McClatchey et al. 2009 [31]	100 children (age 6–16)	Quasi experimental design	UCLA PTSD	PTSD-symptoms	.08	.33	63	.73	−.05	52	.08	.27
	IIB	TG = 46 CG = 54	Evaluation: post treatment	EGI	Childhood Traumatic Grief	.00	.73	77	.90	−.02	51	.01	.50
“Writing for recovery” (CG) no treatment	Kalantari et al. 2012 [28] IB	61 children (age 12–18) TG = 29 CG = 32	RCT Evaluation: 1 week post treatment	TGIC	Traumatic grief	.00	1.26	90	.03	−.39	65	.00	1.21
Family-intervention (CG) no treatment	Black & Urbanowicz 1985 [40]; 1987 [25] IIB	83 children (age 0–16) TG = 38 CG = 45 45 families TG = 21 CG = 24	RCT Evaluation: 1 year post treatment	Clinical Interview	Behavior Sleep Depressed parent	na na na	na na na	na na na	na na na	na na na	na na na	.05 .09 .01	(.21) (.21) (.33)
					Talk about dead parent	na	na	na	na	na	na	.04	(.26)
				Rutter A Rutter A	Restless Nail-biting	na na	na na	na na	na na	na na	na na	.01 .03	(.34) (.28)
					School problems	na	na	na	na	na	na	.10	(.19)
Family-intervention (CG) no treatment	Black & Urbanowicz 1987 [25] IIB	73 children (age 0–16) TG = 38 CG = 35 39 families TG = 21 CG = 18	RCT Evaluation: 2 years post treatment	Clinical Interview	Behavior Talk dead parent	na na	na na	na na	na na	na na	na na	.09 .04	(.28) (.24)
					School Health	na na	na na	na na	na na	na na	na na	.03 .04	(.28) (.39)
“Parent Guidance Program” (CG) telephone monitoring intervention	Christ et al. 2005 [26] IA	104 families with children (age 7–17) TG = 79 CG = 25	RCT Evaluation: 8 and 14 months after parent’s death	CDI SEI STAI-S STAI-T CBCL-soc	Depression Self-Esteem State anxiety Trait anxiety Social competence	.00 .00 .00 .00 .29	.56 .64 .89 .61 .17	71 74 81 73 57	.03 .21 .12 .87 .27	.48 .29 .35 .04 −.31	69 61 64 51 62	.53 .36 .12 .31 .32	.14 .28 .44 .43 .36
				CBCL-bprob	Behavior problem	.17	.16	59	.80	−.07	53	.69	.26
				POPM-tot	Perceived Parenting	.06	.25	60	.31	−.22	59	.11	.37
“The Family Bereavement Program” (first version) (CG) delayed treatment	Sandler et al. 1992 [32] IB	72 families with 72 children (age 7–17) TG = 35 CG = 37	RCT Evaluation: post treatment	CRPBI PRS PRS	Par. warmth Grief discussion Par. Support	.00 .78 .00	.97 .07 .88	83 53 81	.25 .00 .11	.22 −.70 −.31	59 76 62	.03 .03 .01	.50 .62 .83
“The Family Bereavement Program” (revised version) (CG) self-study program	Sandler et al. 2003 [33] IA	156 families TG = 90 CG = 66 244 children (age 8–16) TG = 135 CG = 109	RCT Evaluation: Posttest and 11 months post treatment	Posttest									
				Comp. GLESC Comp. Comp. AIS TAS SPPC MCPCS CBCL CBCL	Pos. parenting Negative events Ment. health Positive coping Inhibition Neg. thoughts Self-esteem Control belieifs Internalizing Externalizing	na na na na na na na na na na	na na na na na na na na na na	na na na na na na na na na na	na na na na na na na na na na	na na na na na na na na na na	na na na na na na na na na na	.00 .03 .01 .02 .01 .78 .37 .72 .03 .11	.58^a^ .43^a^ .50^a^ .30^a^ .48^a^ .05^a^ .19^a^ .06^a^ .41^a^ .28^a^
				11-mth									
				Comp. GLESC Comp. Comp. AIS TAS SPPC MCPCS CBCL CBCL	Pos. parenting Negative events Ment.health Positive coping Inhibition Neg. thoughts Self-esteem Control belieifs Internalizing Externalizing	na na na na na na na na na na	na na na na na na na na na na	na na na na na na na na na na	na na na na na na na na na na	na na na na na na na na na na	na na na na na na na na na na	.03 .11 .10 .20 .06 .18 .16 .00 .61 .19	.39^a^ .32^a^ .32^a^ .18^a^ .39^a^ .29^a^ .27^a^ .40^a^ .10^a^ .24^a^
“The Family Bereavement Program” (CG) self-study program	Schmiege et al. 2006 [37] IA	156 families TG = 90 CG = 66 244 children (age 8–16) TG = 135 CG = 109	RCT Evaluation: 3 and 11 months post treatment		3-months								
				CMAS-R	Anxiety Girls Anxiety Boys	.08^a^ .17^a^	.32^a^ .23^a^	59 57	.32^a^ .04^a^	.20^a^ .38^a^	56 61	.41^c^ .25^c^	.11 −.13
				CDI	Depression Girls Depression Boys	.10^a^ .19^a^	.30^a^ .22^a^	58 56	.28^a^ .37^a^	.21^a^ .17^a^	56 55	.58^c^ .98^c^	.11 .06
				YSR	Externaliz. Girls Externaliz. Boys	.03^a^ .08^a^	.39^a^ .30^a^	61 58	.09^a^ .05^a^	.34^a^ .38^a^	59 61	.36^c^ .50^c^	.08 −.03
				CBCL	Intrenaliz. Girls Internaliz. Boys	.00^a^ .00^a^	.74^a^ .48^a^	70 63	.01^a^ .00^a^	.53^a^ .69^a^	65 69	.88^c^ .39^c^	.19 −.16
				CBCL	Externaliz. Girls Externaliz. Boys	.00^a^ .01^a^	.56^a^ .43^a^	65 62	.07^a^ .00^a^	.37^a^ .57^a^	60 66	.43^c^ .44^c^	.23 −.12
					11-months								
				CMAS-R	Anxiety Girls Anxiety Boys	.02^a^ .01^a^	.43^a^ .44^a^	62 62	.80^a^ .01^a^	.05^a^ .48^a^	51 63	.06^c^ .73^c^	.36 .02
				CDI	Depression Girls Depression Boys	.02^a^ .07^a^	.41^a^ .32^a^	62 59	.55^a^ .05^a^	.12^a^ .37	53 60	.16^c^ .65^c^	.28 −.01
				YSR	Externaliz. Girls Externaliz. Boys	.08^a^ .27^a^	.33^a^ .19^a^	59 55	.89^a^ .13^a^	-.03^a^ .30^a^	51 58	.03^c^ .45^c^	.36 −.08
				CBCL	Intrenaliz. Girls Internaliz. Boys	.00^a^ .01^a^	.80^a^ .47^a^	72 63	.01^a^ .00^a^	.57^a^ .63^a^	66 67	.93^c^ .58^c^	.20 −.10
				CBCL	Externaliz. Girls Externaliz. Boys	.00^a^ .02^a^	.55^a^ .42^a^	65 62	.32^a^ .00^a^	.20^a^ .69^a^	56 69	.11^c^ .86^c^	.40 −.22
“The Family Bereavement Program” (CG) self-study program	Luecken et al. 2010 [29] IA	139 children TG = 78 CG = 61 (age 8–16)	RCT Evaluation: 6 years post treatment	Cortisol	Cortisol level before and after a conflict discussion task	na	na	na	na	na	na	.03^b^	.39^b^
“The Family Bereavement Program” (CG) self-study program	Sandler et al. 2010 [34] IA	156 families TG = 90 CG = 66	RCT Evaluation: Post-test, 11 months and 6 years post treatment		Post (3-months)								
				TRIG IGTS	Present grief Intrusive grief	.19^a^ .16^a^	-.16^a^ .17^a^	54 55	.09^a^ .50^a^	-.23^a^ .09^a^	57 53	.72^c^ .43^c^	.05 .09
		244 children (age 8–16) TG = 135 CG = 109			11-months								
				TRIG IGTS	Present grief Intrusive grief	.69^a^ .00^a^	.05^a^ .47^a^	51 63	.87^a^ .12^a^	-.02^a^ .21^a^	51 56	.88^c^ .06^c^	.08 .27
					6-years								
				TRIG IGTS	Present grief Intrusive grief	.00^a^ .00^a^	.73^a^ 1.30^a^	70 82	.00^a^ .00^a^	.63^a^ 1.08^a^	67 78	.75^c^ .03^c^	.14 .21
“The Family Bereavement Program” (CG) self-study program	Sandler et al. 2010 [35] IA	140 families TG = 78 CG = 62	RCT Evaluation: 6 years post treatment	DISC Compsite	Mental Disorder Externalizing Internalizing	na na na	na na na	na na na	na na na	na na na	na na na	.28^b^ .02^b^ .57^b^	na^b^ .31^b^ na^b^
		218 children TG = 116 CG = 102		RSE PERI BDI	Self-esteem Demoralization Depression	na na na	na na na	na na na	na na na	na na na	na na na	.01^b^ .03^b^ .04^b^	.40^b^ .42^b^ .40^b^

*Note:* TG = Treatment Group; CG = Control Group. Effect size is presented in Cohen’s d and Common Language effect size (CL). The CL effect size indicates that after controlling for individual differences, the likelihood in percent that a person scores a different observed measurement for Mean 1 than for Mean 2. For categorical data ϕ is presented. Numerical data in the publications have been used or recalculated when possible, na = not available, e.g. numbers are missing or calculating not possible based on information in the publication

*Abbreviations: AIS* active inhibition scale, *BID* bellevue index of depression, *CAS* child assessment schedule, *CBCL* child behavior checklist, *CDI* children’s depression inventory, *CMAS-R* children’s manifest anxiety scale-revised, *Comp* composite scale, *CRPBI* children’s reports of parental behavior inventory, *DISC* the diagnostic interview schedule for children, *EGI* the extended grief inventory, *GLESC* general life events schedule for children, *IGTS* intrusive grief thoughts scale, *MCPCS* multidimensional measure of children’s perceptions of control scale, *PERI* psychiatric epidemiology research interview, *POPM* perception of parenting measure, *PRS* parent report scale, *RSE* Rosenberg self-esteem scale, *SEI* self-esteem inventory – short form, *SPPC* self perception profile for children, *STAIC* state-trait anxiety inventory for children, *STAIY* state-trait anxiety inventory for youth, *TAS* threat appraisal scale, *TGIC* traumatic grief inventory for children, *TRIG* Texas Revised Inventory of Grief (Present), *UCLA PTSD* University of California–Los Angeles Post-Traumatic Stress Disorder Reaction Index for the DSM-IV for Children

^a^Due to data presented in article calculated as independent t-test, effect size d_s_

^b^Cohen’s d and *p*-value as reported by the authors of the study in reference. Only findings with *p* ≤ .05 were reported

^c^Due to data presented in article calculated as independent t-test at each time-point (post, follow-up respectively), when controlled for no statistical significant difference in pre-rating

#### Large effects

There were two studies with strong evidence (from robust studies, see definition in Table 1, Grade criteria) that showed *large effects* between groups: for children’s traumatic grief [28]; and parent’s feelings of being supported [32].

#### Medium effects

Four studies showed *medium effects* between groups. Two studies with strong evidence showed medium effects for the parents: for parental warmth [32]; positive parenting [33]; parent’s mental health [33]; and for grief discussions in the family [32]. The following studies with fairly strong evidence showed medium effects: for children’s traumatic grief symptoms [31]; restlessness [40]; and children’s health [25]. One study with fairly strong evidence showed medium effects for parental depression [40].

#### Small effects

Some studies showed *small effects* between groups. The following studies with strong evidence showed small effects: for children’s symptoms of intrusive grief [34]; children’s PTSD symptoms [31]; self-esteem [26, 33, 35]; anxiety [26]; anxiety (girls) [37]; depression (girls) [37]; behaviour problems [26]; social competence [26]; externalizing [33, 35]; externalizing (girls) [37]; internalizing [33]; internalizing (girls) [37]; cortisol level before and after a conflict discussion task [29]; negative events [33]; negative thoughts [33]; control beliefs [33]; positive coping [33]; inhibition [33]; perceived parenting [26]. One study with strong evidence showed small effects for parent’s depression [35]; mental health [33]; demoralization [35]; and positive parenting [33]. The following studies with fairly strong evidence showed small effects: for children’s behaviour problems [25, 40]; sleep problems [40]; nail-biting [40]; talking about the dead parent [25, 40]; and school problems [25, 40].

#### No effects and negative effects

There were a few studies that failed to reveal any effect on measures at any of the post-test or subsequent follow-up test periods. With “No effect” we mean studies where the between group effect size were on Cohen’s d between 0.00 and 0.19 and the effect size calculated as Phi between 0.00 and 0.09. The following studies with strong evidence showed no effects on depression [26] and present grief [34]. One study did not show effects for the subgroup boys on the measures anxiety, depression, internalizing and externalizing [37].

Finally one study showed a small but negative effect for boys’ externalizing behaviour (−0.22), which means that the reduction of externalizing behaviour in boys 11 months post intervention was less in the intervention group than in the control group [37].

## Discussion

The aims of this article were to systematically review empirical studies about effective methods of support for children when a parent or caregiver dies and secondly, to identify gaps in the research. Seventeen studies were included in the review. The included studies were mainly randomized controlled studies, with the exception of two studies, one of which was a quasi-experimental study and the other study employed a pre-post-test design. Thirteen studies provided strong evidence with regards to the quality grading criteria, three provided fairly strong evidence and one provided weaker evidence.

In this review we found large as well as moderate and small between group effects for children and their caregivers. There were effects on children’s grief symptoms, health, behaviour and self-esteem, as well as effects on parenting factors and caregiver’s mental health. There were effects from group interventions directed at children [28], family interventions [25, 29, 32–35, 37, 40], parental guidance [26] and camp activities for children [31].

There were studies that did not show effects on some measures, on depression, present grief, and boy’s anxiety, depression, internalization and externalization. The latter results indicate a need to pay attention to possible gender differences. However, it should also be noted that several of the studies in the review consisted of small numbers of participants, indicating that there is a risk that in some cases there might actually have been a difference between the intervention and control group, which may not have been detected due to the fact that samples were too small to find statistically significant differences when the effect sizes were small. It is also important to keep in mind that most of the included interventions were primary or secondary preventive in nature. That is, they sought to prevent the development of an illness or disease before it even occurred or lower the impact if indeed it already had occurred [57], and thus effect sizes could be expected to be small, but nevertheless remain important for a large group of children [58].

The overall results suggest that even relatively brief supportive interventions can prevent children from developing more severe problems after the loss of a parent [34, 35]. The randomized controlled studies of “The Family Bereavement Program” stand out among the included studies, as the intervention has been evaluated several times, with different outcomes and longitudinally (6 year follow-up period) [27, 29, 30, 34, 35, 41]. After the first included effect study that was published in 1992, the support program has been subsequently revised and refined. The program consists of a total of 14 sessions, including separate groups for caregivers, children and adolescents; joint activities for children and their caregivers; and individual family meetings [59]. The studies concerning “The Family Bereavement Program” from the year 2003 and onwards concern the same version of the support program whose effects have been evaluated from different perspectives. The evaluations of the program also include fidelity of program implementation, assessed as attendance and implementation of the items described in the manuals [33]. The results showed positive effects for both children and caregivers. Studies of the program indicated that some children and families may require more intensive interventions [35, 41] or additional support [38] as the intervention itself is brief.

The results of our review differ from previous reviews that have reported relatively small effect of supportive interventions for bereaved children [15–17]. One reason for the differing results may be that previous reviews often adopted a broader focus by including children who have lost other types of “loved ones”, for example a family member, grandparent, relative or friend [15–17], while this review is focused exclusively on parentally bereaved children. Another reason for the differing results may be that several studies included in previous reviews were excluded in this review for quality reasons, as in some studies the sample was too small for the results to be generalizable. A third reason for the differing results is that some studies of high scientific rigour were published after the previously published systematic reviews. The latest systematic review we found was published in 2010 [17], while eight out of 17 studies in this review were published during the period 2010–2015.

### Implications for practice

The included studies in this review were published within several disciplines, namely psychology, social work, medicine, psychiatry, lending weight to the argument that the subject of support for parentally bereaved children is relevant for a range of different professional groups.

One conclusion from this review of interventions is that there were studies that have shown effects for children and their caregivers. The results indicate that supportive interventions can be directed exclusively to the children or to both the bereaved child and the child’s remaining parent or caregiver. Support for the children’s caregivers can strengthen their own health and their capacity to support their children. A supportive parenting is a protective resource for parentally bereaved children [60]. Previous research indicates that when the bereaved children’s caregivers are supported, they demonstrate an enhanced capacity to support their children [60–62].

At the same time, support also needs to be directed at the children. In the evaluation of a parental guidance program, the remaining parents expressed that they perceived a need for more support directed to their children [26]. In one of the included studies, both children and parents indicated that they wished to discuss grief-related experiences with other people who had similar experiences [32]. Being and connecting with other bereaved children can be helpful for children who attend a support group, as it can help them to feel less isolated and alone [55, 63, 64]. Simultaneous family sessions involving both children and the remaining parent may be an important component in a support program as such sessions are sometimes the first occasion that the parent and children have had the opportunity to sit down together and talk about the loss and their feelings about it [25]. Some children avoid talking about their problems or showing their feelings as they try to protect their remaining parent or other people around them. This can sometimes be misinterpreted as a sign that the child is not affected by the loss [65]. The included effect-evaluated interventions were not sufficient for all children. The majority of intervention programs were brief. Studies indicated that some children may need more intensive support or additional support [31, 35, 36, 38, 41]. Therefore, it is important to reassess children’s further needs for support at the end of an intervention [36].

### Implications for research

Given that there are currently relatively few scientifically rigorous studies in this area, there is a clear need for further research about the effects of support interventions directed at parentally bereaved children. Indeed, there were only 17 studies that met the criteria for this review. All studies, with the exception of one [28], were comprised of studies about English language interventions that were evaluated in the USA or UK (see Table 4). It is evident that there is a need for more effect studies with longer follow-up, with the Family Bereavement Program being a notable exception, as children’s problems can appear later and it may also take time before changes in the participant families stabilize post intervention and have an effect for the children [33]. Furthermore, there is a need for studies with populations sufficiently large enough to make comparisons of the effects for various categories, so that the interventions can be modified to various children’s needs. Some studies for example, showed differences in the efficacy of interventions for children at different ages [35, 41], for girls and boys [26, 33, 35, 37], for mothers and fathers [26] and for children with different levels of problems at baseline [35, 41]. In the majority of included studies the sample were diverse in ethnicity, but did not analyse effects for different ethnic minority groups. The sample sizes of minority groups were too small to allow the testing of program effects for various groups [34]. In the studies, the most common deceased parent was the child’s father with the remaining caregiver the mother. This is consistent with mortality statistic rates as children under the age of 18 are more likely to experience the death of a father than the death of a mother [1].

This systematic review highlights that interventions evaluated with a focus on effects for children have almost exclusively been directed at school age children, while the bereavement research shows increased risks for the youngest children when one or both parents dies [4]. The younger children are especially vulnerable as they are totally dependent on their caregivers. In addition, they often find it more difficult to comprehend what has happened to their deceased parent and what this means [66]. Consequently, development of supportive interventions and evaluation of bereavement interventions for younger children is an important issue for further research. Involving younger children in evaluations of interventions may require innovative methods, where the children are given the opportunity to express themselves in a way that is adapted to their capacity and cognitive development. Such evaluations may also include qualitative interviews where the children can express themselves in their own words or through creative methods such as art or play [63, 67]. Further, children need to be enabled to participate in the research to develop knowledge about their experiences, to explore with children what they themselves perceive as helpful in the grieving process and what kinds of outcome measures are most important from their perspective. For example, few of the outcome measures in the included studies concerned children’s physical health and somatic symptoms, their situation in school and their peer relationships. It is also important that children have the opportunity to be involved in evaluations of support programs as parental reports have a tendency to underestimate children’s problems and report less symptomatology in their children than do the children themselves [68]. Qualitative data from evaluations could also be helpful to identify opportunities to improve current bereavement interventions.

Finally, studies of bereavement interventions for children are more generally focused on children that are living in a nuclear family, where one parent dies and the other parent is the child’s remaining caregiver. However, there are also children who have lived with a single parent who dies, and there are children who lose both their parents through death. These children have to change caregivers and residence. The death of a parent engenders secondary losses that occur as a result of the primary loss. When the child’s only parent or both parents die, the secondary losses are increased, in number and complexity [69]. Therefore, special attention is merited towards these groups of children. One explanation why these children are underrepresented in evaluation studies is that the largest proportion of children in the western world live together with both their parents. It is difficult to conduct evaluation studies with this vulnerable group of children.

## Conclusion

The results of this systematic review of support interventions for parentally bereaved children indicate that relatively brief interventions may help prevent children from developing more severe problems, such as mental health problems and traumatic grief after the loss of a parent. Further research is required including how to best support younger bereaved children. There is also a need for more empirically rigorous studies in this area.

## Abbreviations

AIS: Active inhibition scale
BID: Bellevue index of depression
CAS: Child assessment schedule
CBCL: Child behavior checklist
CDI: Children’s depression inventory
CMAS-R: Children’s manifest anxiety scale-revised
Comp: Composite scale
CRPBI: Children’s reports of parental behavior inventory
DISC: The diagnostic interview schedule for children
EGI: The extended grief inventory
GLESC: General life events schedule for children
IGTS: Intrusive grief thoughts scale
MCPCS: Multidimensional measure of children’s perceptions of control scale
PERI: Psychiatric epidemiology research interview
POPM: Perception of parenting measure
PRS: Parent report scale
RSE: Rosenberg self-esteem scale
SEI: Self-esteem inventory – short form
SPPC: Self perception profile for children
STAIC: State-trait anxiety inventory for children
STAIY: State-trait anxiety inventory for youth
TAS: Threat appraisal scale
TGIC: Traumatic grief inventory for children
TRIG: Texas revised inventory of grief (Present)
UCLA PTSD: University of California–Los Angeles post-traumatic stress disorder reaction index for the DSM-IV for Children
